
JOURNAL INFORMATION
==============================
NLM Title Abbreviation: Pregnancy (Hoboken)
Journal ID: Pregnancy (Hoboken)
NLM Journal ID: 9918994890806676
Journal ID: PMF2

Pregnancy

EISSN: 2997-9684
Publisher: Wiley

ARTICLE INFORMATION
==============================
PMCID: PMC13344669
DOI: 10.1002/pmf2.70160
Article ID: PMF270160
Article version: 1
Subjects: Abstracts, Oral Concurrent Session 5

oral concurrent session 5 Clinical Obstetrics

Abstracts 54 – 63 THURSDAY February 12, 2026 1 PM – 3:30 PM Summit 211 – 214 moderators William Barth, Jr., MD Moeun Son, MD, MSCI

Electronic publication date: 2026 Feb 9
Publication date: 2026 Jan
Volume: 2
Issue: Suppl 1
Electronic Location ID: e70160
Revised 2025 Sep 26; Received 2025 Sep 26; Accepted 2025 Oct 16
Copyright: © 2026 Society for Maternal‐Fetal Medicine. Pregnancy published by Wiley Periodicals LLC on behalf of the Society for Maternal‐Fetal Medicine.
License: This is an open access article under the terms of the http://creativecommons.org/licenses/by/4.0/ License, which permits use, distribution and reproduction in any medium, provided the original work is properly cited.
License URL: https://creativecommons.org/licenses/by/4.0/

Figure count: 0
Table count: 0
Page count: 1

source-schema-version-number: 2.0
cover-date: January 2026
details-of-publishers-convertor: Converter:WILEY_ML3GV2_TO_JATSPMC version:6.8.3 mode:remove_FC converted:07.07.2026

==============================

### Oral Concurrent Session 5

### Clinical Obstetrics

Abstracts 54 – 63

THURSDAY
February 12, 2026
1 PM – 3:30 PM
Summit 211 – 214

MODERATORS
William Barth, Jr., MD
Moeun Son, MD, MSCI
